# The Architecture of the Anbu Complex Reflects an Evolutionary Intermediate at the Origin of the Proteasome System

**DOI:** 10.1016/j.str.2017.04.005

**Published:** 2017-06-06

**Authors:** Adrian C.D. Fuchs, Vikram Alva, Lorena Maldoner, Reinhard Albrecht, Marcus D. Hartmann, Jörg Martin

**Affiliations:** 1 Department of Protein Evolution, Max Planck Institute for Developmental Biology, Spemannstraße 35, 72076 Tübingen, Germany

**Keywords:** proteasome, protein evolution, Anbu, SAXS

## Abstract

Proteasomes are self-compartmentalizing proteases that function at the core of the cellular protein degradation machinery in eukaryotes, archaea, and some bacteria. Although their evolutionary history is under debate, it is thought to be linked to that of the bacterial protease HslV and the hypothetical bacterial protease Anbu (ancestral beta subunit). Here, together with an extensive bioinformatic analysis, we present the first biophysical characterization of Anbu. Anbu forms a dodecameric complex with a unique architecture that was only accessible through the combination of X-ray crystallography and small-angle X-ray scattering. While forming continuous helices in crystals and electron microscopy preparations, refinement of sections from the crystal structure against the scattering data revealed a helical open-ring structure in solution, contrasting the ring-shaped structures of proteasome and HslV. Based on this primordial architecture and exhaustive sequence comparisons, we propose that Anbu represents an ancestral precursor at the origin of self-compartmentalization.

## Highlights

• The crystal structure of the bacterial proteasome homolog Anbu has been solved
• The dodecameric architecture reveals unique features compared with classical proteasomes
• Bioinformatic analysis places Anbu at the root of the proteasome family

## Graphical Abstract

**Figure:**
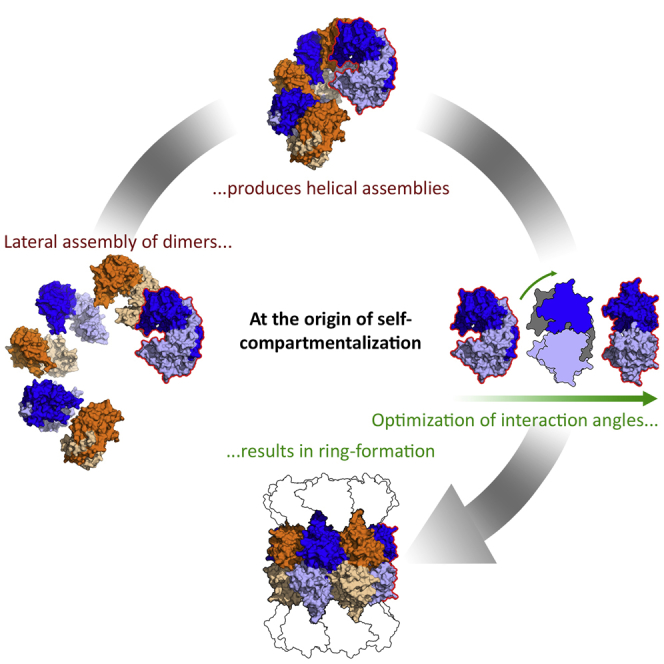

## Teaser

Fuchs et al. present the first experimental study of a new bacterial proteasome homolog called Anbu. The *Pseudomonas aeruginosa* Anbu structure, combined with bioinformatic analyses, allows the authors to draft an evolutionary scenario in which Anbu represents an ancestral proteasome precursor at the origin of self-compartmentalization.

## Introduction

The proteasome is a ubiquitous nano-machine for protein degradation in eukaryotes and archaea (Maupin-Furlow, 2012). It is a ∼670 kDa barrel-shaped complex of four stacked rings (Kish-Trier and Hill, 2013), each composed of seven identical (archaea) or distinct (eukaryotes) subunits. The outer rings consist of catalytically inactive α subunits, whereas the inner rings are composed of proteolytic β subunits. α and β subunits are similar in sequence and structure, and are thought to have emerged by the duplication of a proto-β subunit.

The proteasome can act by itself as a 20S proteasome (Pickering and Davies, 2012a), or its α subunits may interact with various regulators that affect its choice of substrates (Forouzan et al., 2012, Fort et al., 2015). The proteasome's most prominent function, targeted protein degradation, requires interaction with hexameric unfoldases of the AAA+ (ATPase with diverse cellular functions) superfamily (Bar-Nun and Glickman, 2012), which can also act as chaperones on their own (Benaroudj and Goldberg, 2000). In recent years, experiments have also emphasized the significance of the proteasome's ATP-independent functions (Ben-Nissan and Sharon, 2014), such as the degradation of oxidized proteins through interaction with PA28αβ (Pickering and Davies, 2012b) or the degradation of acetylated histones through interaction with PA200 (Qian et al., 2013).

The proteasome is absent from bacteria, barring some branches of Actinobacteria (Lupas et al., 1994, Maupin-Furlow, 2012) and Nitrospirae (De Mot, 2007). While one theory attributes the occurrence of the proteasome in actinobacteria to horizontal gene transfer (HGT) (Gille et al., 2003), another argues that the original proteasome evolved in an ancestral actinobacterium, from where it was inherited by archaea and eukaryotes (Cavalier-Smith, 2006). Both theories, however, assume (Bochtler et al., 1999) that the proteasome as such evolved from its simpler and widely distributed bacterial homolog HslV (heat shock locus V). This homolog, unlike the proteasome, is a homomeric assembly of just two stacked hexameric rings (Bochtler et al., 1997) and thus lacks the antechamber constituted by the α subunits. Despite this, HslV is similar to the proteasome in its ability to interact with an unfoldase of the AAA+ superfamily, HslU, which recognizes intrinsic features of misfolded proteins (Gur et al., 2011). HslU and proteasomal unfoldases, however, belong to different clades of AAA+ ATPases (Ammelburg et al., 2006) and use different interfaces for interaction with their respective proteolytic machinery (Sousa et al., 2000, Yu et al., 2010), suggesting that they were recruited independently. While several studies indicate that HslU is not always bound to HslV (Azim et al., 2005) and possesses chaperone-like activities on its own (Seong et al., 2000), HslV has not been shown to function in the cell on its own in an ATP-independent manner. Unlike the essential, constitutively expressed eukaryotic proteasome, the non-essential heat-shock-induced HslV complements a set of other unrelated self-compartmentalizing proteases, such as FtsH, Lon, and Clp, under stress conditions (Gur et al., 2011, Kanemori et al., 1997).

In 2008, a novel bacterial β subunit homolog, termed Anbu (ancestral β subunit) was identified (Valas and Bourne, 2008). It was proposed that Anbu, not HslV, gave rise to the proteasome, and that this event took place in actinobacteria. This interpretation was, however, questioned as Anbu, unlike other self-compartmentalizing proteases, is not associated with an AAA+ ATPase on the genomic level, but frequently co-occurs in an operon with a transglutaminase, an ATP-grasp protein with putative peptide ligase function, and a unique α-helical protein, Alpha-E (Iyer et al., 2009, Valas and Bourne, 2008), hinting at a specific peptide-synthesis system in which Anbu would act as a peptidase (Iyer et al., 2009).

In this paper, we study the molecular characteristics of Anbu and, based on bioinformatic analysis, its place in proteasomal evolution. We determined crystal structures of two Anbu proteins and could decipher the Anbu structure in solution via small-angle X-ray scattering (SAXS). We find that Anbu forms a dodecameric open-ring assembly that locally resembles the architecture of the self-compartmentalizing proteasome, but is not closed for steric reasons. Based on these findings, we draft a scenario in which the Anbu complex constitutes an evolutionary intermediate at the origin of the proteasome system.

## Results and Discussion

### Anbu Dates Back to the Last Universal Common Ancestor

**Figure 1:**
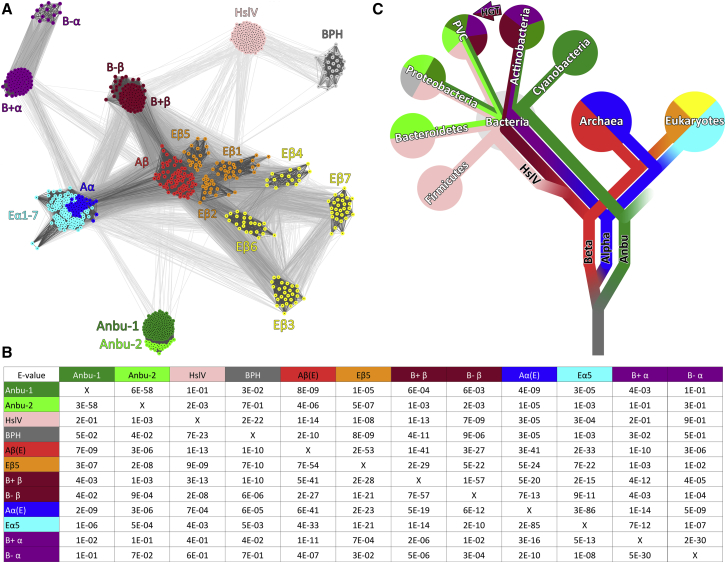
Cluster Map and Evolutionary Scenario for the Origin of Proteasome-like Proteins (A) A cluster map of 969 proteasome-like sequences, with a maximum pairwise identity of 70%, was prepared using CLANS based on their all-against-all pairwise similarities as measured by BLAST p values. Sequences are represented by dots and the line coloring reflects BLAST p values; the darker a line, the lower the p value. Proteasome subunits are abbreviated E for eukaryotic, A for archaeal, and B+/B– for Gram-positive/-negative bacteria. The HslV-derivative BPH (gray cluster) is only found in proteobacteria and therefore appears to play no role in the evolution of the other proteasome homologs. (B) Pairwise similarity of structure-guided sequence alignments. Alignments of selected clusters in (A) were compared and their pairwise similarity expressed as HHalign p values (Soding, 2005). HHAlign uses HMM-HMM (hidden Markov model) comparisons to provide a statistical measure for the relationship between alignments of the indicated proteasome homologs. Thus, this analysis complements the cluster map, where individual sequences are compared. A structural comparison of representative protomers is shown in Table S1. (C) A schematic simplified tree of life (Forterre, 2015) showing the phylogenetic distribution of proteasome-like proteins and our proposed scenario for their evolution in the same colors as in (A). Anbu may have shared a direct ancestry with the proto-β subunit in the last universal common ancestor (LUCA). A subsequent duplication of the proto-β subunit resulted in the emergence of the proteasome, which was then linearly inherited by actinobacteria, archaea, and eukaryotes. HslV evolved by gene duplication of proteasome β at the root of bacteria and replaced the proteasome in many bacterial phyla. Gram-negative bacteria acquired the proteasome by horizontal gene transfer (HGT) from actinobacteria, as previously proposed (De Mot, 2007). Anbu was lost in the common ancestor of eukaryotes and archaea, but was inherited by most bacteria, where it diverged into two subtypes with presumably different substrate specificity (Anbu-1, Anbu-2). Notably, the other proteins of the bacterial Anbu operon are still found sparsely distributed among archaea. The PVC-superphylum comprises Planctomycetes, Verrucomicrobia, and Chlamydiae. See also Figure S1.

To investigate the evolutionary history of Anbu in the context of proteasome evolution, we searched for its homologs in the non-redundant protein sequence database and clustered them by pairwise sequence similarity. In the resulting map, the archaeal α and β subunits form two distinct, but tightly connected, central clusters, which exhibit the highest sequence similarity of all α-β pairs and from which all the other groups radiate (Figure 1A). Also, α subunits of all kingdoms are closer in sequence space to the archaeal β than to any other β sequences and likewise all β subunits are more similar to archaeal α than to any other α sequences (Figure 1B), suggesting that the archaeal proteasome is closest to the original form of the proteasome.

The closest neighbors of these core clusters are the eukaryotic α and β subunits, which are also connected to each other tightly. While the seven α types group together with the archaeal α subunits in a large cluster, the seven β types are more divergent and form distinct subclusters, of which the catalytic ones (β1, β2, and β5) lie closer to the archaeal β subunits, whereas the non-catalytic ones are further removed.

By comparison with their archaeal and eukaryotic counterparts, the bacterial α and β subunits only show residual sequence similarity to each other and are organized into two separate subclusters each (Figures 1A and 1B). While one comprises subunits of Gram-positive actinobacteria and exhibits statistically significant sequence connections to the archaeal core clusters, the other radiates from it and contains subunits of the Gram-negative armatimonadetes, nitrospirae, and verrucomicrobia. This relationship is consistent with the suggestion (De Mot, 2007) that the Gram-negative proteasome may have been acquired by HGT from actinobacteria.

The bacterial proteasome homologs, Anbu and HslV, form two unconnected clusters. HslV sequences group into a single cluster, comprising sequences from almost all bacterial phyla, barring cyano- and actinobacteria. In contrast, Anbu is organized into two closely related subclusters: one comprising sequences from phylogenetically diverse bacteria, mainly from α-/β-/γ-proteo-, cyano-, and actinobacteria (in the following referred to as Anbu-1), and the other (Anbu-2) comprising sequences with a more narrow phylogenetic spectrum (planctomycetes, bacteroidetes, verrucomicrobia, and δ-proteobacteria). Curiously, both Anbu subtypes sometimes co-occur in the same phyla or even in the same organism, e.g., in the α-proteobacterium *Azospirillum brasilense,* indicating that their sequence divergence possibly reflects functional diversification. Furthermore, the distribution of Anbu and HslV in deep-branching bacteria suggests that they were already present in an early bacterium.

As Anbu and HslV are more similar to proteasome subunits of all kingdoms than to each other (Figures 1A and 1B), their evolution is likely not directly linked to each other, but to the proteasome. Notably, HslV exhibits equally significant similarity to the actinobacterial β subunits and to the archaeal β subunits, but only residual similarity to α subunits, suggesting that it arose from a β subunit after the diversification of α and β subunits, very early in bacterial evolution. In contrast, Anbu exhibits only weak similarity to the bacterial α and β subunits, but equally strong similarity to both subunits of the archetypal archaeal proteasome (Figures 1A and 1B). Anbu is thus likely to have either shared a direct ancestry with the proto-β subunit or may even have given rise to it.

These relationships suggest that Anbu and the proteasome were already present in the last universal common ancestor (LUCA) and that the proteasome was linearly inherited by bacteria, archaea, and eukaryotes (Figure 1C). This scenario is, in contrast to previous proposals, not dependent on any HGT events. Notably, this linear inheritance is also supported by the presence of homologs of archaeal proteasome interactors, such as the AAA ATPase PAN or proteasome assembly chaperones (PACs), in actinobacteria (De Mot, 2007, Grana et al., 2009). Since these factors do not occur in a single operon with proteasome α and β in archaea, their co-acquisition through HGT events seems highly unlikely. Furthermore, PAC variants with similarities to both actinobacterial and archaeal sequences are also found sporadically in actinobacteria-related phyla (Hug et al., 2016), such as chloroflexi, which do not contain a proteasome (Figure S1). This strongly suggests that both the proteasome and its associated factors were already established in the LUCA. Since Anbu represents the precursor for the proteolytic core of the proteasome system in this scenario, we decided to tackle it experimentally.

### Anbu Forms a Dodecameric Assembly via the Lateral Association of Six Dimers

**Figure 2:**
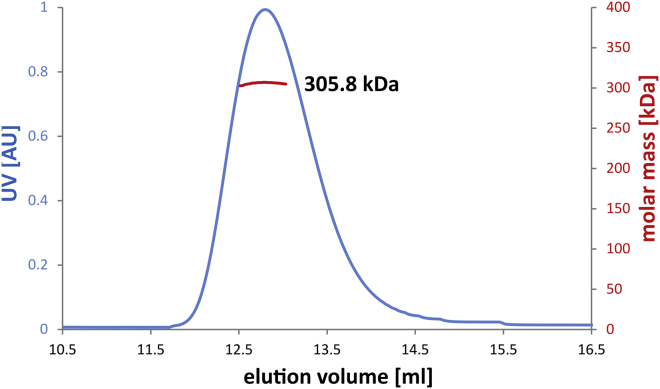
Light-Scattering Profile of Pa-Anbu Pa-Anbu was subjected to a size-exclusion column and the scattering data (red) for the peak area determined in three independent experiments, resulting in 305.8 kDa (shown), 316.1 kDa, and 321.0 kDa, which corresponds to the 11.6–12.2-fold mass of the monomer. See also Figure S2.

For experimental characterization, we recombinantly expressed the Anbu-1 protein from the pathogenic bacterium *Pseudomonas aeruginosa* (Pa-Anbu) in *Escherichia coli*. The purified protein was well folded with a thermal melting point (T_m_) of 55°C. It migrated as a single species in size-exclusion chromatography with an average molecular mass of ∼316 kDa as determined by static light scattering (Figures 2 and S2A), and as a single oligomeric species in native PAGE (Figures S2B and S2C). These results indicated a dodecameric assembly and intuitively suggested an architecture of two stacked hexameric rings similar to HslV.

Crystallization trials yielded a monoclinic crystal form with large cell dimensions that led us to expect about three to four dozens of subunits per asymmetric unit. In the absence of clearly assignable non-crystallographic symmetry and without detectable pseudo-translation, molecular replacement trials with different search models based on various HslV and proteasome β ring structures failed. We therefore prepared a seleno-methionine derivative, which allowed us to solve the structure based on 170 selenium sites belonging to 34 subunits (Table 1).

**Table 1:** Data Collection and Refinement Statistics for Anbu X-Ray Structures

	Native Pa-Anbu	Se-Met Pa-Anbu^L94M/L112M/L228M^	Native Cons-Anbu
Wavelength (Å)	1.0	0.979	1.0
Space group	P2_1_	P2_1_	P4_3_2_1_2
Cell dimensions			
a, b, c (Å)	150.1, 230.0, 172.8	150.1, 230.1, 171.7	170.7, 170.7, 92.10
β (°)	108.0	108.4	
Monomers/ASU	34	34	4
Resolution range data collection (Å)	39.7–3.15 (3.34–3.15)	39.6–2.90 (3.07–2.90)	38.7–2.50 (2.65–2.50)
Completeness (%)	99.2 (97.3)	99.6 (98.2)	99.8 (98.9)
Redundancy	3.47 (3.46)	14.2 (14.1)	10.9 (10.7)
I/σ(I)	9.79 (2.03)	14.3 (2.31)	19.0 (2.50)
R_merge_ (%)	12.3 (74.8)	17.2 (119)	11.7 (99.1)
CC(1/2)	99.3 (70.1)	99.8 (82.2)	99.9 (78.9)
Resolution range refinement (Å)		39.6–2.90 (2.97–2.90)	38.3–2.05 (2.17–2.05)
R_cryst_ (%)		19.8 (35.6)	18.1 (34.0)
R_free_ (%)		21.8 (39.9)	22.2 (40.7)
RMSD			
Bond angles (°)		1.45	1.49
Bond lengths (Å)		0.012	0.012
Ramachandran statistics (%)		91.1/8.9/0	93.1/6.9/0
PDB ID		5LOX	5LOY

Values in parentheses refer to the highest-resolution shell. The Ramachandran statistics show the percentage of residues in favored/allowed/other regions. RMSD, root-mean-square deviation.

**Figure 3:**
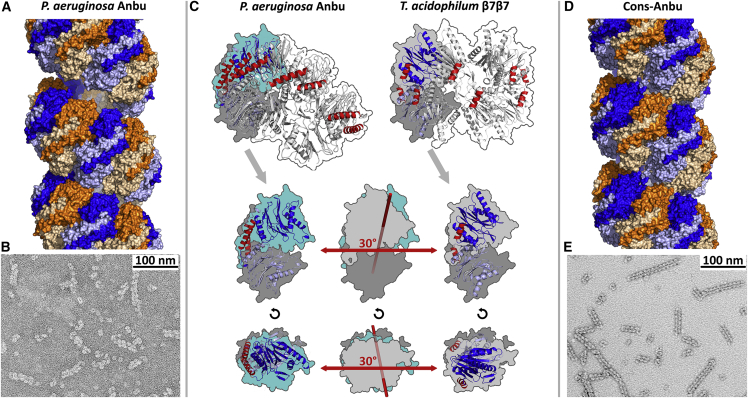
Crystal Structures and EM Micrographs of Pa-Anbu and Cons-Anbu (A and D) About 2.5 turns of the continuous helices are shown for each crystal structure. The dimeric subunits of the helices are alternatingly colored orange and blue, with the lower protomers in lighter shades and the upper protomers in darker shades. Adjacent dimers were crosslinked in the preparation of the EM samples (B and E). (C) Comparison of the geometry of Anbu dimers with the corresponding dimers in proteasomal β rings. The dimers are shown in the context of the Anbu helix or β ring (top row), and isolated in a view orthogonal to the helix or ring main axis (side view, middle row), and in a view along the main axis (top view, bottom row). The lower protomers (dark gray) of the dimers are aligned in their orientation, highlighting the different orientation of the upper protomer (cyan for Anbu, light gray for proteasomal β), totaling in an angular difference of 30°. The respective axis of this rotation is indicated in the middle panels in a superposition of the outlines, which indicate the components of the rotational difference orthogonal and parallel to the main axis. The C-terminal helices of Anbu and proteasome β are highlighted in red.

**Figure 4:**
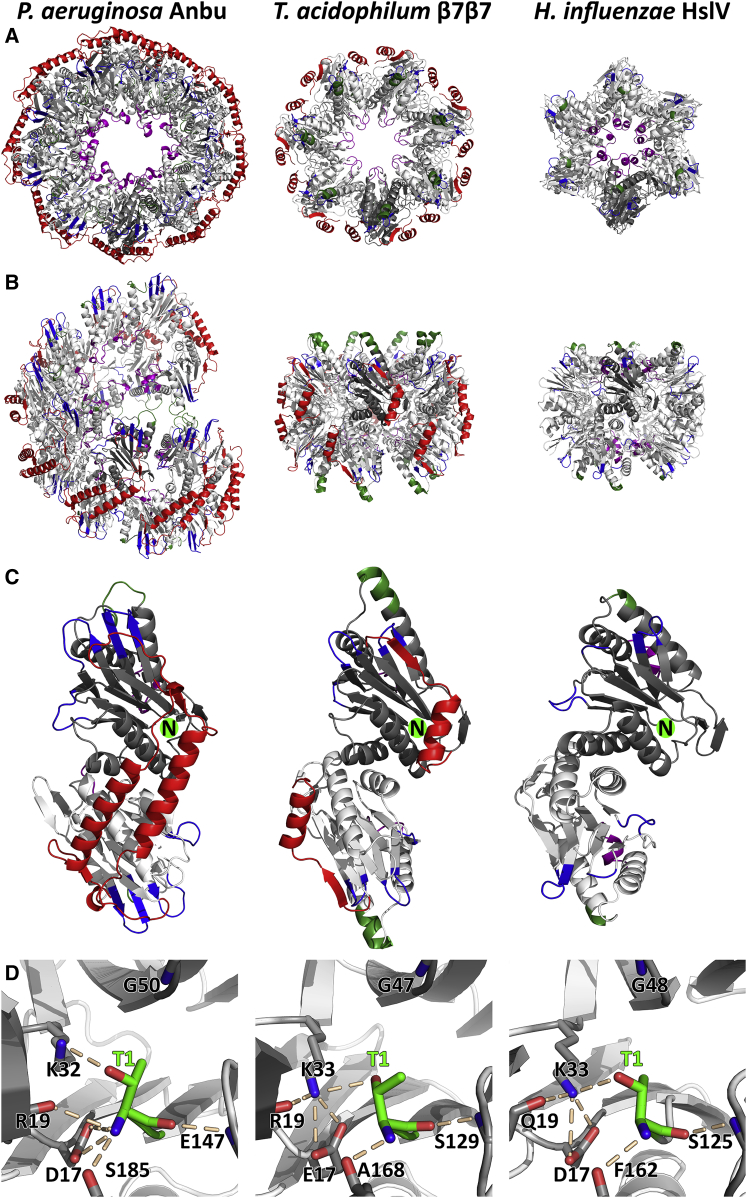
Structural Comparison of Proteasome Homologs (A–C) Shown are top (A) and side views (B), as well as dimers (C) of a helical section of the Pa-Anbu crystal structure, proteasome β7β7 (*T. acidophilum*, PDB: 1PMA; Lowe et al., 1995), and HslV (*H. influenzae*, PDB: 1G3K; Sousa et al., 2000). Divergent sequence elements are highlighted according to the alignment in Figure 5: the extended C-terminal region in Pa-Anbu and proteasome β (red); the gate-keeping (Park et al., 2013) pore loop, which is elongated in Pa-Anbu (purple); the main interface between proteasome β and α subunits, which is present in unstructured form in Pa-Anbu, but not in HslV (green); extended loop regions in Pa-Anbu that could possibly serve as a binding motif (blue). N termini are indicated by green spheres in the upper subunits in (C). (D) Comparison of the active sites, highlighting the catalytically important residues (Groll et al., 1999). The reaction requires the T1 hydroxyl group to be deprotonated by the T1 backbone amino group. The resulting K32/K33-stabilized T1 oxyanion can subsequently attack the substrate peptide bond, resulting in a G47/G48/G50-stabilized oxyanion intermediate. Although relative distances and orientations between these residues show some variability between the three crystal structures, the general mechanism appears to be conserved in the three homologs.

**Figure 5:**
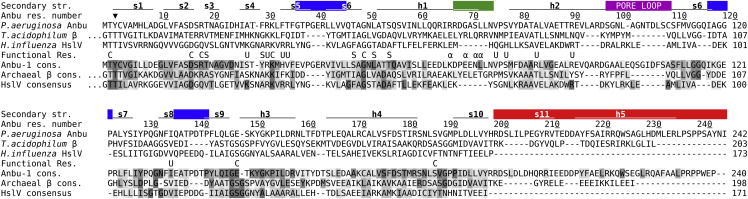
Structure Based Sequence Alignment The color scheme is as in Figure 4. The degree of conservation is indicated by shades of gray in the consensus sequences of the respective proteins. Important functional residues are marked by C (catalytic center), S (substrate specificity pocket S1; Huber et al., 2012), U (HslU interface in HslV), and α (α interface in proteasome β). The arrowhead indicates the cleavage site of the pro-peptide. The *H. influenzae* protein is an unusual HslV representative as it lacks this pro-peptide.

Unexpectedly, these 34 subunits in the asymmetric unit constitute the repeating unit of a continuous helix spanning the crystal (Figure 3A). The individual subunits are essentially all in the same conformation, assuming the same fold as the proteasomal β subunit (Figure 4C). Moreover, a comparison of the active sites shows the catalytic threonine and most other catalytically important residues in essentially the same conformation between Anbu, proteasomal β, and HslV, which suggests the same proteolytic mechanism for all three proteins (Figure 4D). Within the helix, the subunits are arranged in 17 dimers of opposing protomers, distributed over two unequal helical turns. Locally, the subunits are assembled analogous to the β subunits in the double-β ring. However, a prominent difference to the latter lies in the extensive contacts formed between the two protomers within the Anbu dimers. Via an extended C-terminal α-helical segment, Anbu forms a dimeric antiparallel coiled-coil interaction across this subunit interface (Figures 4 and 5), yielding a 3-fold increased interface area for this dimer compared with the respective subunits in the proteasome. Consequently, the intra-dimer interface is virtually invariant and does not exceed 0.4 Å root-mean-square deviation (RMSD) in a superposition of all 17 dimers. This is contrasted by a high degree of variability in the lateral interface between the dimers, enabling the formation of the irregular helix with its two unequal turns that differ in the number of subunits and in the rise per turn: A superposition of all possible pairs of neighboring subunits yields RMSD values of up to 1.6 Å. To elucidate the importance of the stability and integrity of the dimer interface, we prepared a Pa-Anbu deletion mutant with the C-terminal helix truncated to the length of the respective helix in β subunits (Pa-Anbu^Δ227−242^), which would be too short to form an interaction across the dimer interface. Indeed, the mutant protein was unable to assemble into complexes (not shown), substantiating an assembly pathway based on the lateral association of stable dimers. This proposed assembly pathway is fundamentally different to that of the archaeal and eukaryotic proteasome, in which assembly of the β rings depends on preassembled α rings as scaffolds (Kunjappu and Hochstrasser, 2014). A pathway employing stable dimers of opposing subunits could thus reflect an ancient trait to form stable proto-β double rings before the emergence of the α subunits.

### The Shape of the Anbu Subunits Determines Helical Geometry

To find a structural rationale for the unexpected tendency to form helical assemblies, we analyzed the geometry of the basic Anbu dimers in superimpositions to β subunits. Compared with the relative orientation of two opposing subunits in the proteasomal β rings, the two protomers of the Anbu dimers are inclined at a different angle (Figure 3C). The angular difference totals to ∼30° and can be decomposed into two components. The first and larger component is equivalent to a closing hinge motion about an axis perpendicular to the main axis of the ring or helix, yielding a more compact dimer. The second component is equivalent to a shearing motion between the two protomers about an axis parallel to the main axis. While this second component is more subtle, it appears to be sufficient to prevent the assembly into closed rings and drive the crystallization of helices.

As continuous helices obviously do not reflect the native dodecameric assembly, we employed electron microscopy (EM) for its visualization, but could not detect well-defined particles in negative-stain images, neither rings nor helices. We then reasoned that the native Anbu particles might potentially be stabilized, and thus visualized, by inter-dimer crosslinks. To this end, we introduced cysteine residues in juxtaposed positions of adjacent dimers (Pa-Anbu^A53C/N132C^), and promoted disulfide bond formation with Cu-phenanthroline. The EM images of crosslinked mutant proteins, however, showed elongated helices reminiscent of those seen in the crystal structure (Figure 3B), suggesting that closed rings indeed do not exist in solution.

### The Crystal Structure of a Designed Anbu Protein

To test the general validity of our findings, we sought to determine the structure of a second Anbu protein. We attempted to express Anbu from several other organisms but did not succeed in obtaining soluble and folded protein. Therefore, we resorted to a different strategy and designed an Anbu-1 consensus protein (Cons-Anbu), an artificial protein with a sequence corresponding to the most frequently used amino acids in the individual positions of the sequence alignment of all members of the Anbu-1 cluster (Figure 1A). The concept of consensus protein design has emerged as a promising tool in the engineering of stable proteins by making use of evolutionary information embedded in protein sequences (Porebski and Buckle, 2016). Cons-Anbu was indeed highly soluble and even exceeded the thermostability of Pa-Anbu with a T_m_ of 80°C. Crystallization trials yielded a tetragonal crystal form with two dimers in the asymmetric unit (Table 1). Around the crystallographic 4-fold screw axis, these two dimers assemble into a helix with eight dimers per turn (Figure 3D). The dimers superimpose closely on those of Pa-Anbu with an RMSD of 1.0 Å, and the only noteworthy difference between the two structures lies in the geometry of the helices, which is highly regular in the consensus structure. Also for Cons-Anbu, we did not observe well-defined particles in negative-stain EM, but could visualize elongated helices upon cysteine crosslinking (Cons-Anbu^A53CN133C^) as for Pa-Anbu (Figure 3E).

### Anbu Forms an Open Ring of Defined Geometry in Solution

**Figure 6:**
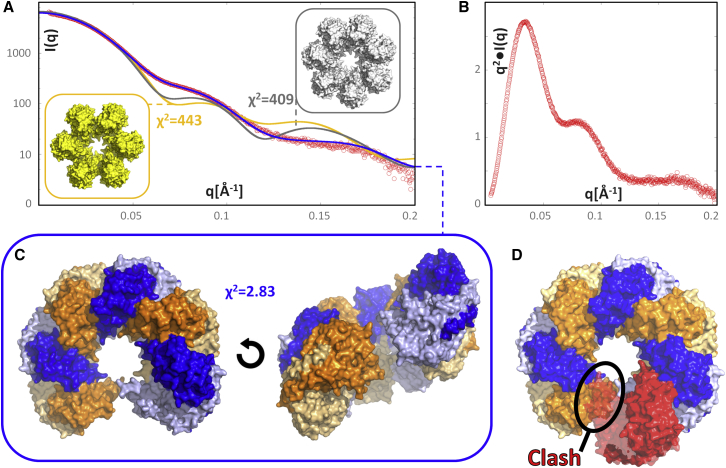
The Solution Structure of the Anbu Complex Determined by SAXS (A) Experimental SAXS data (red) are plotted together with the theoretical profiles of the refined helical complex of six dimers (blue) and the best-fitting models of closed rings with six (yellow) and seven (gray) dimers with the resulting χ^2^ values. (B) The Kratky plot with characteristic shoulders of multi-domain particles, converging to the q axis at high q, indicating low flexibility. (C) Top and side view of the refined solution structure with the subunits colored in shades of blue and orange as in Figure 3.(D) Association of a seventh dimer (red) would result in a steric clash with the first dimer.

As the structure of the native dodecamer still remained elusive, we decided to study Pa-Anbu in solution via SAXS and obtained a SAXS profile of a well-folded oligomeric particle with low intrinsic flexibility as judged from the Kratky plot (Figure 6B). We initially tried to fit the profile with modeled closed rings of six and seven Anbu dimers with different diameters, but could only obtain reasonable fits when using helical segments of six dimers from the Pa-Anbu crystal structure (Figure 6A), clearly indicating that the solution structure is not a closed ring. However, a helical fragment with open ends poses the question of how the assembly process could be terminated after the association of six dimers. We were hoping to find the answer by refining the solution structure in a simple rigid-body approach. Based on six rigid Anbu dimers, we systematically constructed helical segments of different geometries, starting from the geometry of a continuous helix as in the crystal structure. Within each model, the same relative coordinate transformation was applied between the six dimers, yielding symmetric helical segments. We thus constructed a large set of such models, by varying the three Eulerian angles of the rotational component in 1° steps, based on different translations, sampling a conformational continuum of open-ring structures. When these models were fit to the SAXS data, the best agreement was found for models within a small range of parameters, for which the best model with a χ^2^ = 2.83 is depicted in Figure 6. This refined solution structure is an open ring resembling a helical turn with a rise per subunit that is too low to be continued into a second turn; the gap between the first and sixth dimer is too tight for the association of another subunit. As this model, owing to the simple modeling procedure of the refinement approach, is perfectly symmetric, we attempted to refine it further by using established SAXS quaternary structure modeling software without symmetry restraints. However, while these attempts reproducibly resulted in similar overall geometries, their fits to the SAXS profile yielded worse χ^2^ values. These results suggest that the interfaces between the six Anbu dimers are indeed mostly identical in solution, substantiating the fully symmetric open-ring model of the initial approach as the relevant solution structure. Interestingly, functional split-ring structures and spiral architectures have recently been also described for AAA+ ATPases (Glynn and Chien, 2016, Yokom et al., 2016).

### The Active Site Differs between Anbu-1 and Anbu-2 but Shares Common Traits with the Proteasomal β Subunit

**Figure 7:**
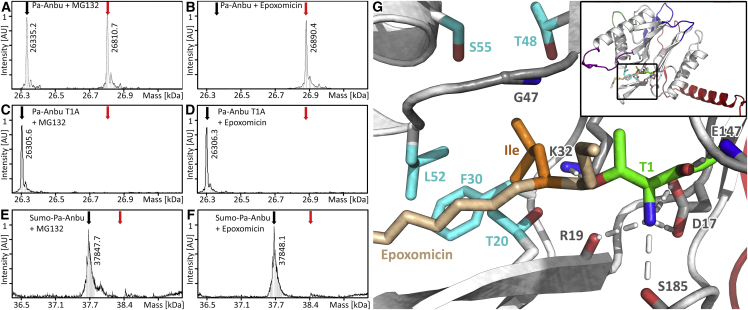
Mass Spectrometric Analysis Shows the Binding of Epoxomicin and MG132 to Anbu (A–F) Pa-Anbu (A and B), Pa-Anbu^T1A^ (C and D), and Sumo-Pa-Anbu (E and F) were incubated with the proteasome-specific inhibitors MG132 or epoxomicin. The resulting protomer masses were determined via mass spectrometry and are shown in comparison with the theoretical masses of the conjugated (red arrow) and unconjugated (black arrow) protomers. See also Figure S3. (G) Model of the Pa-Anbu catalytic center in complex with epoxomicin. The protein backbone is shown as cartoon (white) with the catalytic T1 (green), as well as other residues thought to be crucial for catalysis (gray), and the residues forming the S1 pocket (cyan) represented as sticks. Hydrogen bonds to T1 are indicated by dotted white lines. The structure of the inhibitor epoxomicin, the binding of which to Anbu resulted in poorly diffracting crystals, is taken from the structure of the conjugated *S. cerevisiae* β5 subunit (PDB: 1G65; Groll et al., 2000), which was superimposed on the Pa-Anbu catalytic center with a root-mean-square deviation (RMSD) of 0.67 Å. For simplicity, only the epoxomicin backbone (pale orange) and the terminal leucin-sidechain at P1 (orange) are shown.

The comparison of Anbu with the proteasomal β subunit reveals a highly similar active site geometry with essentially all conserved catalytic residues in place (Figure 4D). While Anbu does not possess a propeptide, in contrast to most β subunits and HslV (Figure 5), its catalytic threonine is exposed at the N terminus in crystal structures and mass spectra (see below), indicating removal of the start-Met by methionine aminopeptidase in vivo (Xiao et al., 2010). In the proteasome, the hydroxyl group of this threonine is deprotonized by the N-terminal amino group and thereby enabled to perform a nucleophilic attack at the peptide bond (Marques et al., 2009). To test this functionality in Pa-Anbu, we assayed Thr-1 binding to the proteasome-specific inhibitors epoxomicin and MG132 via mass spectrometry. To date, no cellular targets, aside from the active eukaryotic proteasome subunits, are known for epoxomicin, since its reaction mechanism requires both activated threonine hydroxyl and amino groups and appropriate positioning by the proteasome substrate binding channel (Huber et al., 2015). We found that both MG132 and epoxomicin were efficiently bound to wild-type Pa-Anbu (Figures 7A and 7B), but not to Pa-Anbu with mutated Thr-1 (Pa-Anbu^T1A^; Figures 7C and 7D) or modified N terminus (Sumo-Pa-Anbu; Figures 7E and 7F). In agreement, tandem mass spectrometry spectra of AspN-digested Pa-Anbu unambiguously confirmed Thr-1 as the site of epoxomicin modification, indicating that this residue indeed acts as an N-terminal nucleophile in Pa-Anbu (Figure S3).

**Figure 8:**
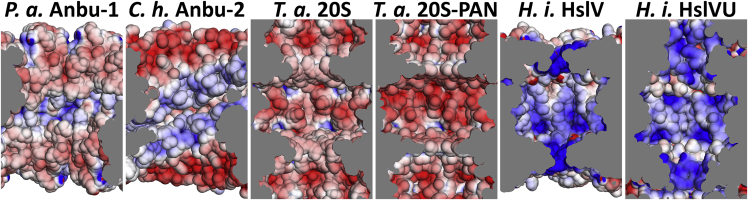
Comparison of Inner Cavities of Proteasome Homologs Shown are *H. influenza* HslV in the presence (PDB: G3I) or absence (PDB: G3K) of the AAA interactor HslU, the *T. acidophilum* proteasome in the presence (PDB: 3IPM) or absence (PDB: 1PMA) of the AAA interactor PAN (PDB: 1PMA), Pa-Anbu, and a *C. hutchinsonii* Anbu-2 model. Poisson-Boltzmann electrostatic potentials (±5 kT/e) are plotted on the surface of the cut-open rings using the Adaptive Poisson-Boltzmann Solver (Baker et al., 2001), with negative potentials in red and positive potentials in blue. The *C. hutchinsonii* Anbu-2 cavity was prepared by homology modeling with Pa-Anbu as a template.

Interestingly, when we tested an Anbu-2 version from *C. hutchinsonii* for this activity, we found the protein unmodified by both inhibitors (data not shown). A similar preference for these inhibitors was observed for various proteasome β subunits and ascribed to their different substrate specificities. These are primarily governed by the composition of the so-called S1 pocket, which binds the side-chain N-terminal to the scissile bond (P1), positioning the latter for cleavage (Huber et al., 2012). The three catalytic eukaryotic β subunits can be distinguished by using different residues in the corresponding positions, most importantly residue 45, and their consistent activity against acidic (*S. cerevisiae* β1 S1 pocket: T20-T31-R45-A49-T52), spacious basic (*S.c.* β2 S1 pocket: S20-C31-G45-A49-T52-E53), or hydrophobic (*S.c.* β5 S1 pocket: A20-V31-M45-A49-C52) peptide substrates (Marques et al., 2009). Anbu was found to be inactive against a standard set of these substrates (see STAR Methods) under numerous conditions tested, which could possibly be ascribed to the requirement for activators or post-translational modifications which were absent in our experiments. However, the Pa-Anbu S1 pockets superimpose exceptionally well with those in the proteasome, revealing a relatively narrow, amphiphilic S1 pocket, in which F30 and L52 form a hydrophobic patch, whereas the moderately hydrophilic T20, T48, and S55 are oriented for hydrogen bonding to various positions in P1. Remarkably, while these S1 pocket residues are highly conserved in Anbu-1 (Figure 5), all Anbu-2 sequences are seen to have an entirely different S1 pocket (*C. hutchinsonii* Anbu S1: I20-A29-R46-R50-T53). Its highly basic characteristic potentially explains why this Anbu-type is incapable of binding MG132 and epoxomicin, which contain hydrophobic residues at P1. Since only S1 pockets with hydrophobic properties are found in archaeal β, bacterial β, and HslV sequences, Anbu-2 might represent the first prokaryotic proteasome homolog tuned for acidic cleavage specificity. This is mirrored by the properties of the inner cavity surface, which is more positively charged in Anbu-2 (Figure 8).

Although there are no known interactors, there are two unique structural features on the surface of the Anbu complex that could play a role in the docking of potential cofactors. The most obvious of the two features is the unused oligomerization interface on either end of the open ring, which is almost fully solvent exposed. The second feature concerns the outer rims of the ring, which protrude more prominently toward the outside than in the β rings (blue in Figures 4 and 5). As both these potential binding surfaces form a right angle with respect to each other at either end of the ring, it is tempting to speculate that they might jointly mount a cofactor each on top and on the bottom of the complex.

### The Biological Function of Anbu

What can be discerned from our structural studies about the physiological role of Anbu? The presence of many identical cleavage sites in a ring-shaped structure, as seen here, argues for processivity, a key feature of self-compartmentalizing proteases (Akopian et al., 1997). Substantiating a protease function is the binding of specific proteasome inhibitors which mimic the first steps of the cleavage reaction (Figure 7), and the evolution of different substrate binding pockets in Anbu-1 and Anbu-2. Thus, our characterization agrees with a function of Anbu in protein degradation, making the previously proposed involvement in a peptide-synthesis system (Iyer et al., 2009) less likely. As different proteasome homologs frequently co-occur within a given organism, rather than being mutually exclusive as originally thought (De Mot et al., 1999), their different substrate specificities could provide the cell with a broader functionality, as is accomplished by the presence of alternative proteasome subunits and interactors in eukaryotes (Fort et al., 2015).

While a partner AAA ATPase for Anbu appears to be absent, the proteasome provides a precedent for the exertion of ATP-independent functions, both with non-ATPase interactors and as an uncapped 20S proteasome, which has been implicated in the degradation of oxidized and otherwise damaged proteins (Pickering and Davies, 2012a). While being expressed constitutively in moderate amounts, neither Anbu nor the other operon proteins are essential for bacterial growth under non-stress conditions (Molina-Henares et al., 2010). Their expression does not change under various environmental conditions (Dotsch et al., 2015), including nitrosative stress (Firoved et al., 2004), a situation in which the mycobacterial proteasome is required for resistance to nitric oxide (Darwin et al., 2003). Anbu is upregulated, however, in nitrogen-limited *Pseudomonas putida* cultures (Hervas et al., 2008), suggesting a contribution to a higher protein turnover under these conditions. The activity and active conformation of Anbu could be further regulated by covalent modifications, as is known for the proteasome (Hirano et al., 2016). However, attempts to obtain a different Anbu form by purifying the endogenous protein from *P. aeruginosa* or by co-expression with the other operon proteins were not met with success (not shown). Thus, a key task for the future will be to find physiological substrates and potential interactors.

### Anbu as a Precursor of Self-Compartmentalizing Proteases

Our bioinformatic analysis places Anbu at the very early stages of proteasomal evolution in the LUCA, but it cannot clearly assign the relative ancestry of Anbu and the proto-β subunit. However, our structural characterization suggests an evolutionary scenario in which the Anbu complex represents a precursor of the self-compartmentalizing proteases. Generally, in evolving a homo-oligomeric assembly, two distinct, complementary interaction surfaces have to be established on the protomer. In a first trial, it is highly unlikely that the relative orientation of these interfaces yields either a perfectly straight polymer or a closed ring without helical rise. Most likely, the orientation of the interfaces would yield a curved polymer with non-zero helical rise. However, a helical rise larger than the height of the subunits would potentially yield infinite polymers, which would not be desirable in many cases. If the helical rise per turn is sufficiently small though, the assembly cannot proceed into a second turn, but instead forms a defined oligomer, as seen in Anbu. Such, a primordial Anbu protein or Anbu-like protein had already established the ability to oligomerize into proteasome-like proteolytic chambers in the LUCA. However, its helical geometry was subsequently superseded by the formation of closed ring assemblies that allowed the construction of modular systems such as the proteasome, facilitating the recruitment of further rings of regulatory proteins. A key necessity in this process was presumably the trimming of the C-terminal helices to disrupt the rigid coiled-coil interface of the dimeric subunits. While Anbu was then lost in archaea, a gene duplication of proteasome β led to the emergence of HslV in the common ancestor of bacteria, which subsequently replaced the proteasome in non-actinobacterial strains (Figure 1C). The Anbu proteins we characterized here are presumably direct descendants of the ancient evolutionary intermediate that survived in a functional niche alongside the modular bacterial self-compartmentalizing proteases.

## STAR★Methods

### Key Resources Table

**Table:** 

REAGENT or RESOURCE	SOURCE	IDENTIFIER
**Antibodies**		

Rabbit Anti-Pa-Anbu	Davids Biotechnology	N/A
HRP-coupled goat anti-rabbit IgG	Sigma	Cat#AP307P; RRID:AB_11212848

**Biological Samples**		

*P. aeruginosa* PAO1 genomic DNA	DSMZ	Cat#DSM-22644
*C. hutchinsonii* ATCC 33406 genomic DNA	DSMZ	Cat#DSM-1761

**Chemicals, Peptides, and Recombinant Proteins**		

FastDigest NdeI	Thermo	Cat#FD0583
Fast-digest HindIII	Thermo	Cat#FD0504
FastDigest AgeI	Thermo	Cat#FD1464
FastDigest XhoI	Thermo	Cat#FD0694
1,10-Phenanthroline	Sigma	Cat#131377
Epoxomicin	Apexbio	Cat#A2606
MG132	Merck	Cat#474791
Complete Protease Inhibitor	Roche	Cat#04693116001
Ac-Gly-Pro-Leu-Asp-AMC	Enzo life sciences	Cat#BML-AW9560
Z-Leu-Leu-Glu-AMC	Enzo life sciences	Cat#BML-ZW9345
Suc-Leu-Leu-Val-Tyr-AMC	Enzo life sciences	Cat#BML-P802-0005
Ac-Arg-Leu-Arg-AMC	Enzo life sciences	Cat#BML-AW9785
Boc-Leu-Arg-Arg-AMC	Enzo life sciences	Cat#BML-BW8515
Mca- Ala-Lys-Val-Tyr-Pro-Tyr-Pro-Met-Glu-Dap(Dnp)	Enzo life sciences	Cat#BML-ZW8505-0005
Mca-Ala-Lys-Val-Tyr-Pro-Tyr-Pro-Met-Glu-Dap(Dnp)	GenScript	N/A
H-Val-AMC	Bachem	Cat#I-1385
H-Tyr-AMC	Bachem	Cat#I-1665
H-Thr-AMC	Bachem	Cat#I-1360
H-Pro-AMC	Bachem	Cat#I-1290
H-Phe-AMC	Bachem	Cat#I-1285
H-Asp-AMC	Bachem	Cat#I-1775
H-Ala-AMC	Bachem	Cat#I-1410
H-Ile-AMC	Bachem	Cat#I-1420
TMB	Thermo	Cat#34021

**Critical Commercial Assays**		

EnzCheck Protease Assay	Thermo	Cat#E6638
P-check peptide library	Jena Bioscience	Cat#PP-408

**Deposited Data**		

*T. acidophilum* proteasome structure	Lowe et al., 1995	PDB: 1PMA
*T. acidophilum* proteasome - PAN-N structure	Yu et al., 2010	PDB: 3IPM
*H. influenzae HslV* structure	Sousa et al., 2000	PDB: 1G3K
*H. influenzae HslV* - HslU structure	Sousa et al., 2000	PDB: 1G3I
*S. cerevisiae* proteasome structure	Groll et al., 2000	PDB: 1G65
Se-Met Pa-Anbu L94M/L112M/L228M structure	This study	PDB: 5LOX
Cons-Anbu structure	This study	PDB: 5LOY

**Experimental Models: Organisms/Strains**		

*E.coli* BL21 Gold (DE3)	Thermo	Cat#50-125-348

**Oligonucleotides**		

See Table S2 for primers used in this study		

**Recombinant DNA**		

Plasmid: pET22b Pa-Anbu	This Study	N/A
Plasmid: pET22b Pa-Anbu L94M/L112M/L228M	This Study	N/A
Plasmid: pET22b Pa-Anbu A53C/N132C	This Study	N/A
Plasmid: pET22b Pa-Anbu Δ226-242	This Study	N/A
Plasmid: pET22b Pa-Anbu T1A	This Study	N/A
Plasmid: pET28b Sumo-Pa-Anbu	This Study	N/A
Anbu-cons gene	Eurofins	N/A
Plasmid: pET30b Anbu-cons	This Study	N/A

**Software and Algorithms**		

MPI Bioinformatics Toolkit	Alva et al., 2016	https://toolkit.tuebingen.mpg.de/
PSI-BLAST	Altschul and Koonin, 1998	https://toolkit.tuebingen.mpg.de/psi_blastp
CLANS	Frickey and Lupas, 2004	https://toolkit.tuebingen.mpg.de/clans
PROMALS3D	Pei et al., 2008	http://prodata.swmed.edu/promals3d/promals3d.php
HHfilter	Soding, 2005	https://toolkit.tuebingen.mpg.de/hhfilter
HHalign	Dolinsky et al., 2007	https://toolkit.tuebingen.mpg.de/hhalign
Consensus Maker	HIV sequence database	http://www.hiv.lanl.gov
PyMOL	Schrödinger	v1.8.0.5; https://www.pymol.org/
APBS PDB2PQR	Dolinsky et al., 2007	v2.1; http://www.poissonboltzmann.org/
MODELLER	Sali et al., 1995	v3; https://toolkit.tuebingen.mpg.de/modeller
DaliLite v3	Hasegawa and Holm, 2009	v3.1; http://ekhidna.biocenter.helsinki.fi/dali_lite/start
XDS	Kabsch, 2010	http://xds.mpimf-heidelberg.mpg.de/
SHELXD	Sheldrick, 2008	http://shelx.uni-ac.gwdg.de/
ARP/WARP	Perrakis et al., 1999	http://www.embl-hamburg.de/ARP/
Buccaneer	Cowtan, 2006	http://www.ysbl.york.ac.uk/∼cowtan/buccaneer/buccaneer.html
Coot	Emsley and Cowtan, 2004	https://www2.mrc-lmb.cam.ac.uk/personal/pemsley/coot/
REFMAC5	Murshudov et al., 1999	https://www2.mrc-lmb.cam.ac.uk/groups/murshudov/content/refmac/refmac.html
MOLREP	Vagin and Teplyakov, 2000	http://www.ccp4.ac.uk/html/molrep.html
ScÅtter	Biosis	www.bioisis.net
CCP4 suite pdbset	Winn et al., 2011	http://www.ccp4.ac.uk/html/pdbset.html
FoXS	Schneidman-Duhovny et al., 2016	https://modbase.compbio.ucsf.edu/foxs/
Sasref	Petoukhov and Svergun, 2005	https://www.embl-hamburg.de/biosaxs/sasref.html

### Contact for Reagent and Resource Sharing

Further information and requests for resources and reagents should be directed to and will be fulfilled by the Lead Contact, Jörg Martin (Joerg.Martin@tuebingen.mpg.de).

### Method Details

#### Bioinformatics

To gather sequences of proteasome homologs, we searched the non-redundant protein sequence database at NCBI, comprising either bacterial, archaeal or eukaryotic proteins, employing four iterations of PSI-BLAST (Altschul and Koonin, 1998) at default settings. The following proteins were used as seeds for the first iteration of these searches: *P. aeruginosa* Anbu, *H. influenzae* HslV, *C. metallidurans* BPH, *T. acidophilum* and *M. tuberculosis* proteasome α and β. After each iteration, sequences to be included for the next iteration were manually reviewed. The sequences resulting from each of the searches were filtered down to a pairwise sequence identity of 90% using HHfilter in the MPI Bioinformatics Toolkit (Alva et al., 2016). The sequences in these reduced sets were next clustered by their all-against-all pairwise BLAST P-values in CLANS (Frickey and Lupas, 2004), to identify and remove incomplete or unrelated sequences. Sequences contained in the individual clusters of the resulting cluster maps were subsequently aligned using PROMALS3D (Pei et al., 2008), based on homologs with three-dimensional structures. The alignments were refined manually and propeptides as well as inserts of unusual lengths were removed. To further decrease redundancy, for the purpose of creating a global cluster map of proteasome homologs, we filtered these alignments down to a maximum pairwise identity of 70% using HHfilter. Next, we pooled together all sequences in these alignments and clustered them in CLANS to generate the cluster map of proteasome homologs shown in Figure 1A. Clustering was done to equilibrium in 2D at a BLAST P-value cutoff of 1e-20 and the final cluster map was made by showing all connections with a P-value better than 1e-09. The sequences in the 70% set were also used as input for HHalign (Soding, 2005) comparisons at default settings and to generate majority-rule consensus sequences with the consensus maker tool (http://www.hiv.lanl.gov). These consensus sequences were subsequently used for the manually refined PROMALS3D alignment shown in Figure 5. Clustering was done to equilibrium in 2D at a BLAST P-value cutoff of 1e-15 and the final cluster map was made by showing all connections with a P-value better than 1e-10.

The PAC cluster map (Figure S1) was generated using an approach similar to the one described for the cluster map of proteasome homologs; the PSI-BLAST search was seeded with an alignment of archaeal PAC2-like (Kusmierczyk et al., 2011) sequences.

Structures were visualized using PyMOL v1.8.0.5, electrostatic potentials calculated at ± 5 kT/e with the APBS plugin and PDB2PQR (Dolinsky et al., 2007) using default settings. The *C. hutchinsonii* Anbu-2 homology model was generated with MODELLER (Sali et al., 1995), using the Pa-Anbu structure as template. DaliLite v3 (Hasegawa and Holm, 2009) was used for calculating structural alignments and similarity scores of the monomeric structures.

#### Cloning, Expression and Protein Purification

*P. aeruginosa* PAO1 and *C. hutchinsonii* genomic DNA were used to amplify and clone genes PA1733 and CHU_3460 into the pET22b expression vector using NdeI and HindIII restriction sites. The Pa-Anbu construct was the basis for “round-the-horn” (Hemsley et al., 1989) site-directed mutagenesis to obtain Pa-Anbu^A53C/N132C^ for intersubunit crosslinking, a Pa-Anbu^Δ226-242^ deletion mutant to investigate the stabilizing function of the C-terminus and a Pa-Anbu^T1A^ mutant to substantiate T1 as catalytic residue. It was also used to generate a Pa-Anbu^L94M/L112M/L228M^ mutant for selenomethionine (Se-Met) labeling (Se-Met Pa-Anbu) via fusion-PCR. The Sumo-Pa-Anbu fusion was generated by cloning Pa-Anbu into a pET28b vector containing the *S. cerevisiae* sc288 Smt3p (Sumo) gene with AgeI and XhoI restriction sites. The Anbu-Con sequence was derived from an alignment of all available Anbu-1 sequences with a maximum sequence identity of 90%. The synthesized gene (Eurofins) was cloned into the pET30b vector using NdeI and HindIII restriction sites. The Cons-Anbu^A53CN133C^ mutant for intersubunit crosslinking was generated via site-directed mutagenesis. *E. coli* BL21 gold cells (Thermo) were transformed with the respective plasmids and grown at 25°C in M9 minimal medium supplemented with 50 μg/ml Se-Met, Leu, Ile, Phe, Thr, Lys and Val for Se-Met labeling, or in lysogeny broth (LB) for all other purposes. Protein expression was induced at an optical density of 0.4 at 600 nm with 0.5 mM isopropyl-β-D-thiogalactoside. Cells were harvested after 16 h, lysed by French press, and cleared from cell debris by ultracentrifugation. Soluble proteins were purified using two sequential anion-exchange columns, QHP (GE Healthcare) at pH 6.2 (20 mM MES/NaOH, 1 mM dithiothreitol (DTT), 0-500 mM NaCl) and MonoQ (GE Healthcare) at pH 8.8 (20 mM Tris/HCl, 1 mM DTT, 0 - 500 mM NaCl), followed by gel size-exclusion chromatography (Sephacryl S-300 HR, GE Healthcare) at pH 7.5 (20 mM Tris/HCl, 50 mM NaCl). Peak fractions containing the respective proteins were pooled, concentrated, supplemented with 15% glycerol, flash frozen in liquid nitrogen and stored at -80°C.

#### Biochemical and Biophysical Methods

For electron microscopy (EM), disulfide crosslinks were generated by incubation of 140 μg/ml protein (5 μM subunits) with 1.5 mM Cu(II)-Phenanthroline for 5 min at room temperature, followed by addition of 100 mM EDTA. Glow-discharged carbon-coated grids were incubated with 0.1 mg/ml protein suspension, stained with 1% uranyl acetate and examined with a FEI Tecnai G2 Spirit BioTwin transmission EM at 120kV.

Thermal denaturation curves to monitor protein stability were recorded by circular dichroism spectroscopy at 220 nm using a JASCO J-810 spectropolarimeter.

Static light-scattering experiments were performed with 100 μl protein at concentrations of 0.5 mg/ml, 1 mg/ml or 2 mg/ml in 30 mM MOPS/NaOH (pH 7.2), 150 mM NaCl, using a Superdex S200 10/300 GL gel size-exclusion column (GE Healthcare) coupled to a miniDAWN Tristar Laser photometer (Wyatt) and a RI-2031 differential refractometer (JASCO). Data analysis was carried out with ASTRA V software (Wyatt).

Proteolytic activity was assayed in 20 mM Tris/HCl pH 7.5, 50 mM NaCl, supplemented with c0mplete protease inhibitor (Roche) without EDTA, which does not inhibit proteasome-like proteases. Enzyme (10 nM subunits when concentration was fixed) and fluorogenic substrates (50 μM each) were incubated at 30°C, and fluorescence changes recorded continuously for 2 h (Synergy H4 microplate reader, Biotek). Assay parameters were modified by varying pH (4.5 - 9.0), temperature (25 - 60 °C), salt (50 - 500 mM NaCl, 0 - 50 mM KCl, 0 - 5 mM MgCl_2_/CoCl_2_/CaCl_2_) and enzyme concentrations (1 - 1000 nM). Assayed substrates included BODIPY-casein (Thermo), Ac-Gly-Pro-Leu-Asp-AMC, Z-Leu-Leu-Glu-AMC, Suc-Leu-Leu-Val-Tyr-AMC, Ac-Arg-Leu-Arg-AMC, Boc-Leu-Arg-Arg-AMC, Z-Gly-Gly-Leu-AMC (Enzo life sciences), Mca- Ala-Lys-Val-Tyr-Pro-Tyr-Pro-Met-Glu-Dap(Dnp) (GenScript), H-Val-AMC, H-Tyr-AMC, H-Thr-AMC, H-Pro-AMC, H-Phe-AMC, H-Asp-AMC, H-Ala-AMC, H-Ile-AMC (Bachem) and the P-check peptide library (Jena Bioscience).

Native Western blots were performed using the Novex 4-16% Bis-Tris protein gels (Thermo) according to manufacturer's instructions. Pa-Anbu was detected using Pa-Anbu specific antibody raised in rabbit (Davids Biotechnology) and visualized with HRP-coupled goat anti-rabbit IgG (Sigma) and TMB (Thermo).

#### Mass Spectrometry

For liquid chromatography mass spectrometry (LCMS) measurements, 1 mg/ml (38 μM subunits) Pa-Anbu, Pa-AnbuT1A or Sumo-Anbu were incubated with either a 20x molar excess of epoxomicin or a 2x molar excess of MG132 for 48 h at 4°C (20 mM HEPES/NaOH pH 7.5, 150 mM NaCl). To determine the masses of Pa-Anbu protomers, desalted samples were subjected to a Phenomenex Aeris Widepore 3.6 μm C4 200 Å (100 x 2.1 mm) column using an Agilent 1100 HPLC, eluted with a 30- 80% H_2_O/acetonitrile gradient over 15 min at a flowrate of 0.25 ml/min in the presence of 0.05% trifluoroacetic acid, and analyzed with a Bruker Daltonik microTOF. Eluted proteins were ionized at 4500 V and mass to charge (m/z) ratios determined in the range 800 – 3000. Data processing was performed in Compass DataAnalysis 4.2 and the m/z deconvoluted to obtain the protein mass via MaxEntropie.

To validate the Pa-Anbu N-terminus as site of epoxomicin modification, the epoxomicin treated Pa-Anbu sample was also used for AspN digestion and subsequent LC-MS/MS analysis. Desalted samples were subjected to an EasyLC nano-HPLC (Proxeon Biosystems) coupled to an LTQ Orbitrap XL (Thermo Scientific) at a flow rate of 200 nl/min, and eluted with a segmented gradient of 8 - 26 - 40 - 64 - 72% H_2_O/acetonitrile in the presence of 0.1% formic acid. Full scans were acquired at a resolution of 60,000. The target values were set to 5000 charges for the LTQ (MS/MS) and 10^6^ charges for the Orbitrap (MS), respectively. The MS data were processed with MaxQuant software suite v.1.5.2.8 (Cox and Mann, 2008). MS/MS spectra were searched with the Andromeda module (Cox et al., 2011) against a database consisting of 4,313 protein entries from E. coli, 285 commonly observed contaminants and Pa-Anbu. Protein N-terminal acetylation and methylation, methionine oxidation, and epoxomicin modification were set as variable modifications. Initial precursor mass tolerance was set to 4.5 parts per million (ppm), and at the fragment ion level 0.5 dalton (Da) was set for CID fragmentation. False discovery rates were estimated by the target/decoy approach (Elias and Gygi, 2007) and set to 1%. MS/MS spectra were obtained for both oxidized and non-oxidized versions of the modified N-terminal Pa-Anbu peptide (TYCVAM[ox]HLA). In these spectra, the epoxomicin moiety was represented as the peptide IITL with N-terminal acetylation and methylation, and the morpholino adduct between epoxomicin and Pa-Anbu Thr-1 as C_3_H_6_O.

#### Crystallization, Data Collection and Crystal Structure Determination

For crystallization, native Pa-Anbu, the Se-Met derivative of Pa-Anbu^L94M/L112M/L228M^ (Se-Met Pa-Anbu) and native Cons-Anbu were concentrated to 3.7, 11.2 and 20 mg/ml, respectively, in 50 mM NaCl, 20 mM Tris-HCl pH 7.5. Initial screening of conditions was performed in 96-well sitting-drop plates, with drops containing 300 nl of protein solution and 300 nl of reservoir solution, and a reservoir of 50 μl. For Se-Met Pa-Anbu and Cons-Anbu, crystallization conditions were further optimized in hanging drop setups, with drops containing 2.5 μl of protein solution and 2.5 μl of reservoir solution, and a reservoir of 500 μl in EasyXtal plates (Qiagen). The crystals used in the diffraction experiments grew within two days in 100 mM sodium acetate pH 4.4, 1.5 M sodium nitrate for Pa-Anbu, 100 mM sodium acetate pH 4.8, 1.2 M sodium nitrate for Se-Met Pa-Anbu, and 100 HEPES-NaOH pH 7.0, 600 mM NaF for Cons-Anbu. Prior to loop-mounting and flash-cooling in liquid nitrogen, the crystals were briefly transferred into a droplet of reservoir solution supplemented with either 25 % ethylene glycol (Pa-Anbu, Se-Met Pa-Anbu) or 3.5 M NaCl (Cons-Anbu) for cryo-protection. Data were collected at 100 K and a wavelength of either 1 Å (Pa-Anbu, Cons-Anbu) or 0.979 Å at the Selenium K-edge (Se-Met Pa-Anbu) at beamline X10SA of the Swiss Light Source (Villigen, Switzerland), using a PILATUS 6M hybrid pixel detector (Dectris Ltd.). All data were indexed, integrated and scaled using XDS (Kabsch, 2010), with the statistics given in Table 1.

For Se-Met Pa-Anbu, we employed SHELXD (Sheldrick, 2008) for heavy atom location. As we expected about three to four dozens of subunits with five selenium sites each, we decided to search for 220 heavy atoms. In the 14,344th trial, a solution with a CC All/Weak of 49.2 / 25.8 and CFOM of 75.0 was found, with a sharp drop in peak height after about 160 strong sites. After phasing and density modification with SHELXE, many secondary structure elements could be traced by ARP/WARP (Perrakis et al., 1999), and it became apparent that the asymmetric unit contains 34 subunits, arranged in a double-helix spanning the crystal. The structure was completed by cyclic chain tracing with (Buccaneer, (Cowtan, 2006)), manual modeling (Coot, (Emsley and Cowtan, 2004)), and refinement (REFMAC5, (Murshudov et al., 1999)) with strong NCS restraints. The native Pa-Anbu structure was subsequently solved on the basis of the Se-Met structure. As it did not reveal any conformational differences, but was of lower resolution than the Se-Met strufture, it was not regarded further; We therefore refer to the Se-Met Pa-Anbu structure as the Pa-Anbu structure.

The structure of Cons-Anbu was solved by molecular replacement with MOLREP (Vagin and Teplyakov, 2000), using two consecutive dimers of the Pa-Anbu structure as the search model, and the structural model completed and refined using Coot and REFMAC5. In the Cons-Anbu crystals, the double helix is built around the crystallographic 4_3_ axis with 8 dimers per turn. Refinement statistics are given in Table 1.

#### Small-Angle X-Ray Scattering (SAXS)

SAXS experiments were performed at beamline B21, Diamond Light Source (Didcot, UK), with a X-ray wavelength of 1 Å and a PILATUS 2M detector at a distance of 3.9 m. A sample of 50 μl Pa-Anbu at a concentration of 19 mg/ml was delivered at 20 °C via an in-line Agilent HPLC with a Shodex Kw-403 column and a running buffer consisting of 20 mM TRIS pH 8.0, 150 mM NaCl, 2 mM TCEP and 1% sucrose. The continuously eluting sample was exposed for 300 s in 10 s acquisition blocks, and the data pre-processed using in-house software. Frames recorded immediately before the elution of the sample were used for buffer subtraction. Buffer subtraction and further analysis were performed with ScÅtter version 2.2b. According to the Kratky plot and a Porod exponent of 3.8, Pa-Anbu forms a well-folded particle with low intrinsic flexibility.

For fitting the SAXS profile, models of different geometries were prepared using a dimer from the Pa-Anbu crystal structure and the program pdbset of the CCP4 suite (Winn et al., 2011). Closed 6- or 7-rings of different radii were constructed by placing the dimer at different distances in 1 Å intervals to the origin and applying sixfold symmetry or 51.43° rotations. Helix sections with six or seven dimers were directly cut out of the Pa-Anbu crystal structure.

For the refinement of helical models, the translational components and the three Euler angles of the coordinate transformation from one dimer to the next in the crystal structure were determined. Then, a series of helical models of six dimers was constructed by applying the same transformation five times using pdbset, by varying the translational components and individual Euler angles between different models in 1 Å and 1° steps. The fits of these models to the SAXS profile were assessed using the program FoXS (Schneidman-Duhovny et al., 2016), with the best model resulting in χ^2^=2.83. For further refinement, this best-fitting model was subjected to rigid body modeling in sasref (Petoukhov and Svergun, 2005), with two loose contact restraints between each pair of dimers, i.e. ten restraints in total. Multiple such trials did not improve the fit further and did not change the helical nature of the complex.

### Data and Software Availability

All data were deposited in PDB with the following entry codes: (Se-Met Pa-Anbu^L94M/L112M/L228M^) (Native Cons-Anbu).

## Author Contributions

A.C.D.F., M.D.H., and J.M. designed the research. A.C.D.F., L.M., R.A., and M.D.H. performed the research. A.C.D.F., V.A., M.D.H., and J.M. analyzed the data, A.C.D.F., V.A., M.D.H., and J.M. wrote the paper.

## Supporting Citations

The following references appear in the Supplemental Information: Altschul et al., 1997, Holm et al., 2008, Wei et al., 2012.

## Supplemental Information

Document S1. Figures S1–S3 and Tables S1 and S2

Document S2. Article plus Supplemental Information
