# Supplementary material for: The Architecture of the Anbu Complex Reflects an Evolutionary Intermediate at the Origin of the Proteasome System
Source: Structure. 2017 Jun 6;25(6):834–845.e5. doi: 10.1016/j.str.2017.04.005 (PMC5666114; doi:10.1016/j.str.2017.04.005)
Supplement: Document S1. Figures S1–S3 and Tables S1 and S2 [file mmc1.pdf]

Structure, Volume *25*

Supplemental Information

The Architecture of the Anbu Complex Reflects an Evolutionary Intermediate at the Origin of the Proteasome System

Adrian C.D. Fuchs, Vikram Alva, Lorena Maldoner, Reinhard Albrecht, Marcus D. Hartmann, and Jörg Martin

Supplemental Information

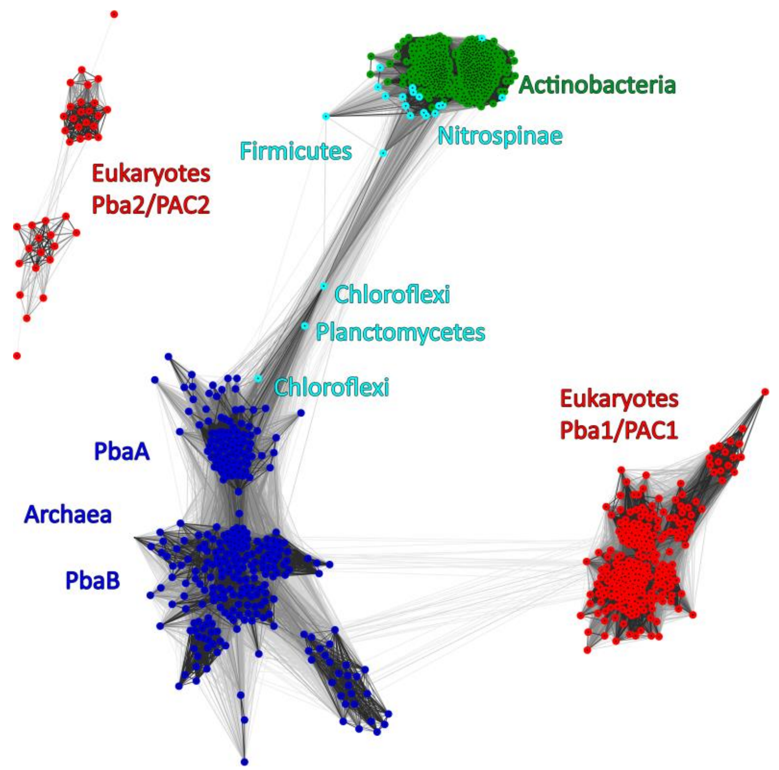

Figure S1, related to Figure 1C. Cluster map of proteasome assembly chaperones (PAC) and proteasome biogenesis associated (Pba) proteins

A cluster map of 1046 PAC and Pba protein sequences, with a maximum pairwise identity of 70%, was prepared using CLANS (Frickey and Lupas, 2004). Sequences are represented by dots and the lines connecting them represent the statistical significance of their similarity; the darker a like, the lower the BLAST (Altschul et al., 1997) P-value. The map shows that the proteasome-associated PACs also occur dispersed in various proteasome-deficient bacteria (cyan) and show similarities to both archaeal (blue) and actinobacterial (green) sequences. This observation is in agreement with our proposal of an acquisition of the actinobacterial proteasome system by linear inheritance (Figure 1C).

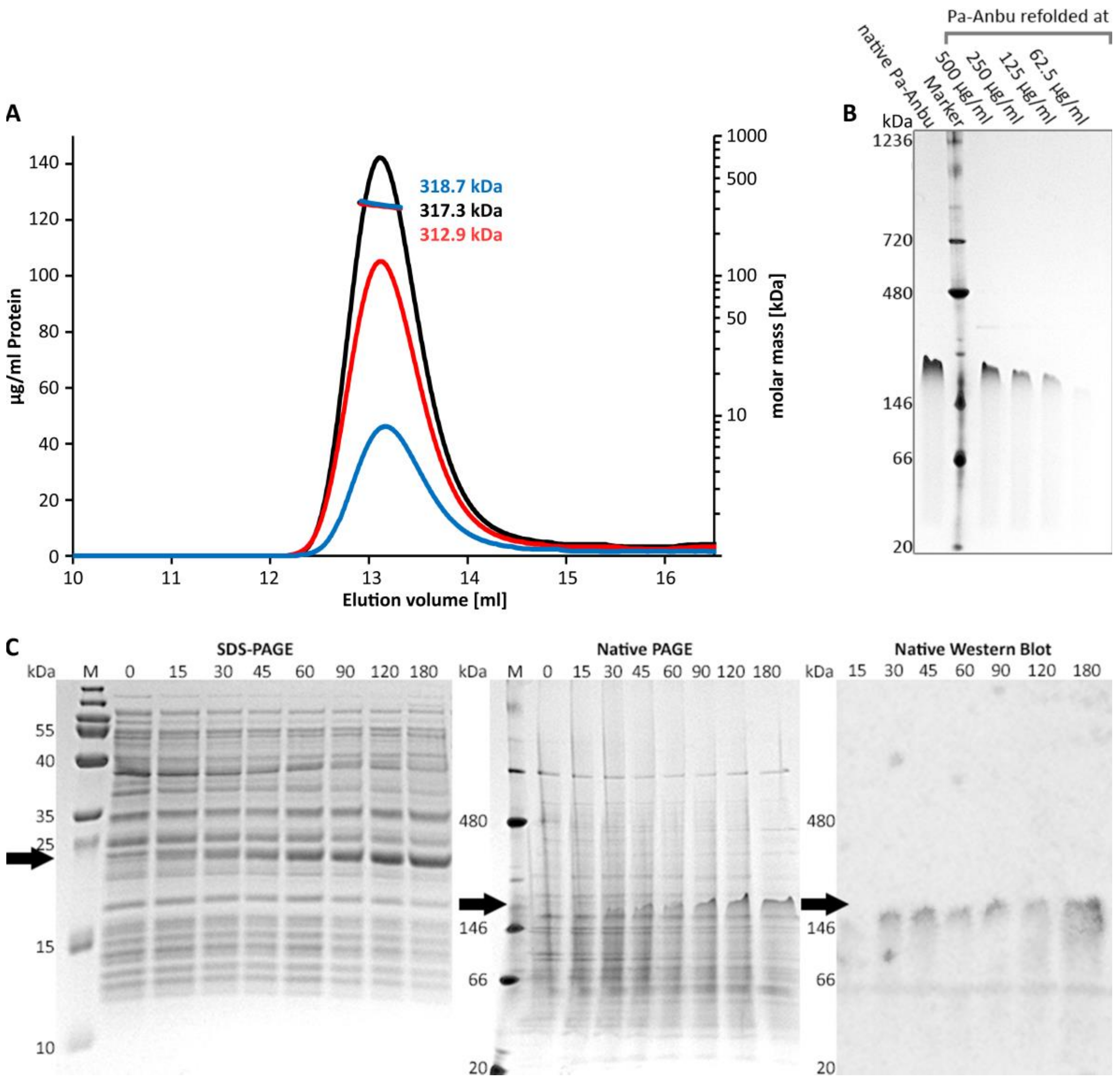

Figure S2, related to Figure 2. Pa-Anbu is a dodecamer in solution.

(A) Light scattering profiles of Pa-Anbu at three different protein concentrations. 100 µl Pa-Anbu at 2 mg/ml (black profile), 1 mg/ml (red) or 0.5 mg/ml (blue) was subjected to a S200 size exclusion column and the mass of the eluted particles in the peak area was analyzed via static light scattering. The protein concentration in the eluted fractions, plotted on the left axis, was determined by integration of the UV signal of all samples.

(B) Native PAGE of refolded Pa-Anbu. Pa-Anbu was denatured in 8 M urea and subsequently refolded at the specified concentrations by dialysis against buffer (20 mM HEPES-NaOH pH 7.5, 150 mM NaCl). The gel shows 10 µl of the refolded soluble fractions, migrating at the same size as native Pa-Anbu (left lane).

(C) Oligomeric state of Pa-Anbu at different expression levels. Pa-Anbu was recombinantly produced via the T7 expression system in *E. coli*. Cells were grown at 37°C in LB medium and induced at an OD of 0.4 with 1 mM IPTG. Protein expression was stopped at the indicated time points (in minutes), and corresponding protein extracts were analyzed by Commassie Blue-stained SDS-PAGE (left panel), native PAGE (middle panel), and Western blot of a native PAGE using anti-Pa-Anbu antibody (right panel).

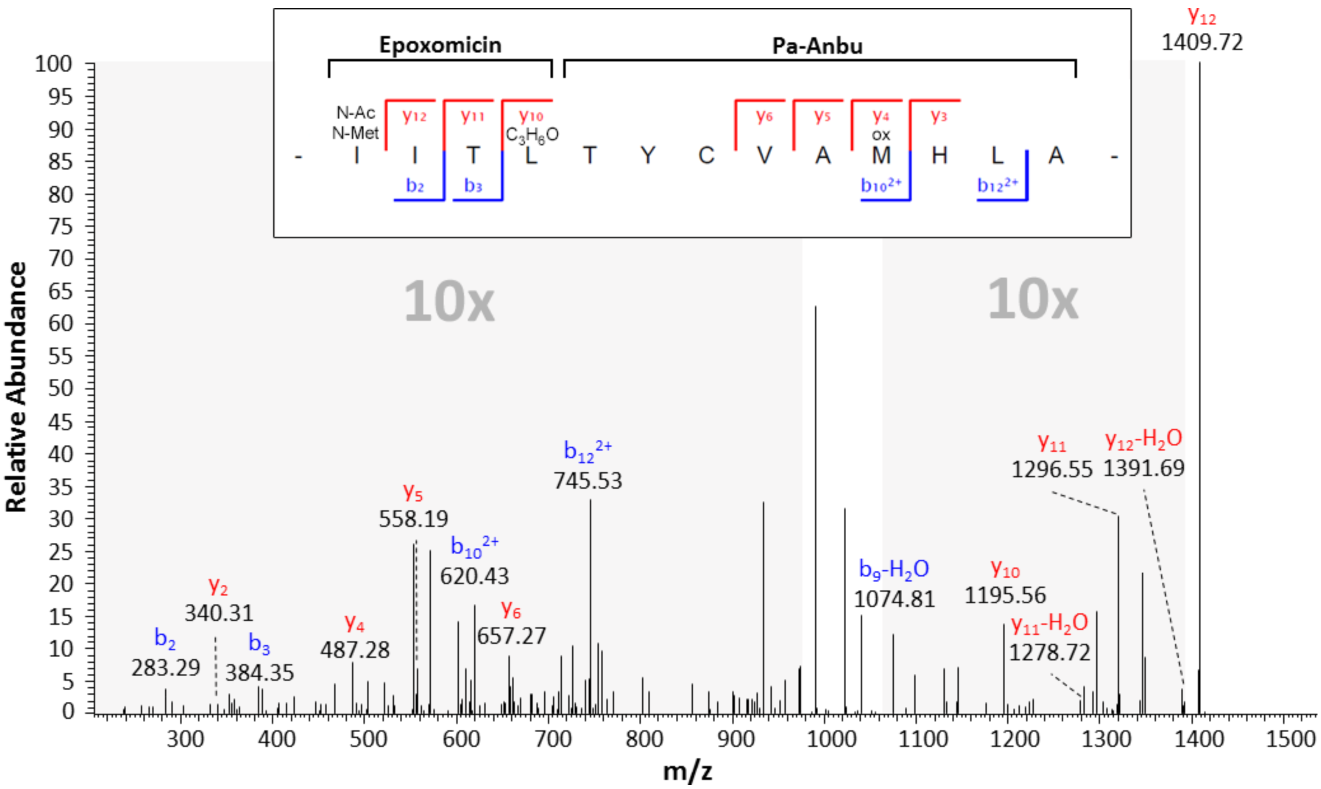

Figure S3, related to Figure 7. MS/MS spectrum of the epoxomicin-modified N-terminal Pa-Anbu fragment after AspN digestion.

Epoxomicin-treated Pa-Anbu (Figure 7B) was digested with AspN and subjected to MS analysis. A mass shift corresponding to the molecular weight of epoxomicin (554.36 Da) was detected only for the N-terminal fragment, but not for other fragments in the digest. The sequence (see inset) of the modified N-terminal fragment was traced via the shown MS/MS-spectrum. For this purpose, the epoxomicin moiety was interpreted as the peptide IITL with N-terminal acetylation and methylation. The morpholino adduct between epoxomicin and Pa-Anbu Thr-1 is represented by its chemical formula, C_3H_6O (Wei et al., 2012). Within the spectrum, b and y series ions are indicated. For better visualization, grey areas in the spectrum were magnified by 10x.

Table S1, related to Figure 1B. Structural comparison of proteasome-type protomers

DALI Z-Score	PA-Anbu	*H. i.* HslV	*T. a.* 20S-β	*M. t.* 20S-β	*T. a.* 20S-α	*M. t.* 20S-α
PA-Anbu	X	16.7	21.3	19.8	18	16.9
H. i. HslV	16.2	X	21.2	21	17.2	15.4
T. a. 20S-β	22	21.3	X	28.4	25.8	22.6
M. t. 20S-β	19.8	21	28.4	X	22	20.8
T. a. 20S-α	17.6	16.9	25.5	22	X	25.4
M. t. 20S-α	16.9	15.4	22.6	20.8	25.4	X

The structural similarity of the monomeric *T. acidophilum* and *M.tuberculosis* proteasome α- and β-subunits, Pa-Anbu and *H. influenzae* HslV in absence of interactors is expressed by DALI Z-scores (Holm et al., 2008). Greater structural similarity results in higher Z-Scores. Comparing HslV und proteasome subunits in presence of interactors results in similar, slightly lower Z-scores.

Table S2, related to the STAR methods key resources table. List of primers used in this study

Primer	Sequence
**Pa-Anbu Forward**	5'-CGATATCATATGACCTACTGTGTCGCG-3'
**Pa-Anbu Reverse**	5'-CGATGCAAGCTTAGATGTTGTACGCCGAGG-3'
**Pa-Anbu$^{1\text{-}108\ L94M}$ Forward**	5'-GATATACATATGACCTACTGTGTCGCGATGCAC-3'
**Pa-Anbu$^{1\text{-}108\ L94M}$ Reverse**	5'-GTCGGTATTGCCGGCCAGATTGCCGCTGTCGCGGCCATCACCTCGCG-3'
**Pa-Anbu$^{100\text{-}244\ L112M/L228M}$ Forward**	5'-GCAATCTGGCCGGCAATACCGACCTGAGCTGTTCCTTCATGGTCGGCG-3'
**Pa-Anbu$^{100\text{-}244\ L112M/L228M}$ Reverse**	5'-GGCCGCAAGCTTAGATGTTGTACGCCGAGGGCGGCGACGGCAGGCGCTCCAGCATGTCGTGCAG-3'
**Pa-Anbu^{A53C} Forward**	5'-Phospho-TGCACCTCGCAATCGGTGATCAAC-3'
**Pa-Anbu^{A53C} Reverse**	5'-Phospho-CAGGTTGCCGGCCGTCTGC-3'
**Pa-Anbu^{N132C} Forward**	5'-Phospho-TGCTTCATCCAGGCCACGCCGG-3'
**Pa-Anbu^{N132C} Reverse**	5'-Phospho-GCCCTGGGGATAGATGCTGTAC -3'
**Pa-Anbu$^{\Delta 226\text{-}242}$ Forward**	5'-Phospho-TAACTGCACGACTTGCTGGAG-3'
**Pa-Anbu $^{\Delta 226\text{-}242}$ Reverse**	5'-Phospho-ACCTGCGCTCCACTGGC-3'
**Pa-Anbu^{T1A} Forward**	5'-Phospho-GCATACTGTGTCGCGATG-3'
**Pa-Anbu$^{1\text{-}108\ L94M}$ Reverse**	5'-Phospho-CATATGTATACTCCTTCTTAAAG-3'
**Sumo-Pa-Anbu Forward**	5'-TCATCTACCGGTGGAACCTACTGTGTCGCGATGC-3'
**Sumo-Pa-Anbu Reverse**	5'-CTAGATCTCGAGTCAGATGTTGTACGCCGAG-3'
**Cons-Anbu^{A53C} Forward**	5'-Phospho-TGCACCACCCAGGCAGTG-3'
**Cons-Anbu^{A53C} Reverse**	5'-Phospho-CAGATTACCTGCGGTCAGC-3'
**Cons-Anbu^{N133C} Forward**	5'-Phospho-TGCTTCATTGAGGCGACCC-3'
**Cons-Anbu^{N133C} Reverse**	5'-Phospho-ACCCGCCGGGTAAATC-3'

All primers were synthesized by Sigma Aldrich.
